# Extrapolating Local Coupled Cluster Calculations toward CCSD(T)/CBS Binding Energies of Atmospheric Molecular Clusters

**DOI:** 10.1021/acsomega.5c04476

**Published:** 2025-09-29

**Authors:** Yosef Knattrup, Jonas Elm

**Affiliations:** Department of Chemistry, 1006 Aarhus University, Langelandsgade 140, 8000 Aarhus C, Denmark

## Abstract

Aerosols are the largest source of uncertainty in modern global radiative forcing modeling. Atmospheric molecular clusters are important intermediates in atmospheric new particle formation (NPF). The evaporation rate of clusters can be calculated using quantum chemical methods, with an exponential dependence on the free energy. Hence, for simulating accurate NPF rates, high-accuracy calculations are needed. We have constructed a versatile benchmark set of 218 conformers of atmospheric molecular dimer clusters consisting of sulfuric acid (SA), formic acid (FA), nitric acid (NA), methanesulfonic acid (MSA), water (W), ammonia (AM), methylamine (MA), dimethylamine (DMA), trimethylamine (TMA), and ethylenediamine (EDA) molecules. Using this test set, we benchmark the local coupled cluster methods, DLPNO–CCSD­(T_0_) and LNO–CCSD­(T), using different basis sets and locality settings, and test extrapolation procedures to the complete basis set (CBS), local approximation free (LAF), and complete PNO space (CPS) limits. The extrapolations are tested against the binding energies of high-level CCSD­(F12*)­(T+)/cc-pVTZ-F12 reference calculations. We find that the LNO–CCSD­(T) methods offer a better accuracy-to-cost ratio for atmospheric molecular clusters than the usually employed DLPNO–CCSD­(T_0_) method. Furthermore, the CBS limit extrapolation using the aug-cc-pVTZ and aug-cc-pVQZ basis sets should be readily attainable for the LNO–CCSD­(T) method on the usually studied cluster sizes (4–8 monomers). Simulating the new particle formation rate of the (SA)_1–4_(AM)_1–4_ and (SA)_1–4_(DMA)_1–4_ systems using the Atmospheric Cluster Dynamics Code, we find an increased sensitivity to the locality settings for larger clusters, but the basis set error is still the most dominant. Hence, simulated cluster formation rates would also benefit from doing LAF extrapolation. Finally, we illustrate the calculations of LNO–CCSD­(T)/CBS binding energies of a large (SA)_15_(TMA)_15_ cluster (300 atoms). Hence, the application of LNO–CCSD­(T) allows for significantly more accurate binding energies of much larger clusters than previously possible.

## Graphical abstract

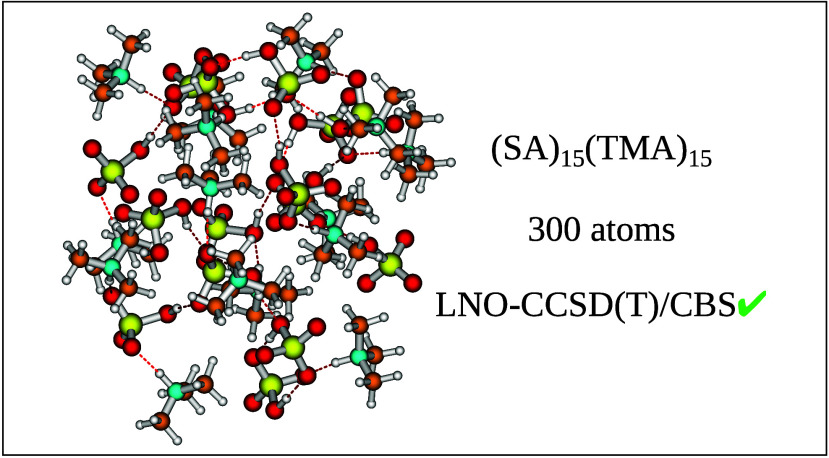

## 1 Introduction

Aerosols are liquid or solid particles suspended in a gas, measuring over 2 nm. They counteract the warming caused by greenhouse gases by cooling the atmosphere.^1^ This cooling effect occurs because aerosols can directly scatter sunlight or act as cloud condensation nuclei (CCN), tiny particles that serve as the starting points for cloud droplet formation.^2^ When more CCN are present, clouds form with a higher number of smaller water droplets, which makes them appear whiter and more reflective.^3^ As a result, these clouds reflect more sunlight, contributing to a cooling effect on the climate. The sixth IPCC report highlights that aerosols remain the largest source of uncertainty in modeling global radiative forcing.^1^ A significant part of this uncertainty comes from gaps in our understanding of the initial formation mechanisms.^4^

Aerosol formation occurs through two processes. Primary aerosol formation happens when aerosols are directly emitted into the atmosphere, such as from volcanic eruptions, sea spray, or dust storms. Secondary aerosol formation occurs when atmospheric gases undergo gas-to-particle conversion to form clusters that can grow into aerosol sizes.^5^ This process is called new particle formation (NPF). The dominant formation mechanism depends on the local environment, but it has been shown that secondary aerosol formation accounts for up to approximately 60% of CCN over Western United States and between 30–40% over mainland Europe.^6^

The NPF process has been strongly linked to the clustering of sulfuric acid (SA) with bases of high abundances such as ammonia (AM)^5,7–11^ or high basicity alkyl-amines such as methylamine (MA), dimethylamine (DMA),^10,12–21^ trimethylamine (TMA)^10,14,16,17,19,20^ and ethylenediamine (EDA).^10,22–25^ Furthermore, species such as nitric acid (NA),^26–37^ formic acid (FA),^34,36,38–42^ methanesulfonic acid (MSA)^43–51^ and water (W)^15,52–66^ have been shown to enhance cluster formation.

The initial secondary formation process can be explicitly simulated as collisions and evaporation events of the monomers and clusters. Such schemes describe the cluster distribution via solutions to the birth-death equations.^67^

1 $$\frac{dc_{i}}{dt}=\overset{\left\lfloor i/2\right\rfloor }{\underset{i=1}{\sum }}s_{j,\left(i-j\right)}c_{j}c_{\left(i-j\right)}+\underset{j}{\sum }\gamma_{\left(i+j\right)\to i}c_{i+j}-\underset{j}{\sum }s_{i,j}c_{i}c_{j}-\overset{\left\lfloor i/2\right\rfloor }{\underset{j}{\sum }}\gamma_{i\to j}c_{i}$$

where *c*_*i*_ is the concentration of cluster *i*, *t* the time, γ_*i*→*j*_ the evaporation of *i* to form *j*, and *s*_*i*,*j*_ the sticking coefficient for sticking-collisions between *i* and *j*. The sticking coefficient is often approximated by kinetic gas theory as the collision coefficient of hard spheres β.

The evaporation coefficients can be calculated using detailed mass-balance under the assumption that the evaporation rate does not change significantly from equilibrium:^68^

2 $$\gamma_{\left(i+j\right)\to i}=s_{ij}\frac{c_{i}^{e}c_{j}^{e}}{c_{i+j}^{e}}=s_{ij}c_{\mathrm{ref}}\exp\left(\frac{\Delta G_{i+j}-\Delta G_{i}-\Delta G_{j}}{k_{B}T}\right)$$

where *c* ^*e*^ is the equilibrium concentration, *c*_ref_ the reference vapor concentration, Δ*G* the binding free energy, *T* the temperature, and *k*_B_ the Boltzmann constant.

The exponential dependence on the free energies requires a high degree of accuracy. The largest error in cluster formation studies has been attributed to the electronic single point energy of the density functional theory (DFT) methods used to calculate the geometries and vibrational frequencies.^12,13,69^ To overcome this problem, a composite scheme where a higher level calculation is used for the electronic energy with the DFT-based geometries and frequencies is often applied.

To obtain accurate cluster distribution dynamics simulations, three major requirements must be satisfied for the calculated free energies: 1) the free energy has to be calculated as accurately as possible to minimize the exponentially growing error in the evaporation rates, 2) the method must be applicable to all compositions and sizes of clusters included in the simulation, as they require the relative free energies to be consistent, and 3) the computational cost must be reasonable as the calculations should be applicable in large screening workflows involving numerous conformers.

Commonly, only clusters containing 4–8 monomers are explicitly simulated, as larger clusters are assumed stable in the simulation and will grow spontaneously from this point to aerosol sizes.^12,13,69^ For these sizes, the NormalPNO DLPNO–CCSD­(T)/aug-cc-pVTZ level of theory^70–75^ has been used as the standard single point correction, as it has been shown to perform excellently in benchmarks.^76–79^

The basis sets by Dunning and co-workers^80–84^ have been developed to smoothly and systematically converge toward the complete basis set (CBS) limit as a function of increasing cardinal numbers. Therefore, by calculating successive cardinal number energies, the energies can be extrapolated toward the CBS limit. Several schemes exist,^85–94^ but most treat the self-consistent field (SCF) and correlation energy separately as they do not converge following the same form.

While a three-point CBS extrapolation scheme^85^ has been used by the Shield group with great success,^36,95,96,96–98^ a systematic benchmark of basis set extrapolation schemes for atmospheric molecular clusters has not been carried out. Likewise, there exists an extrapolation scheme for the pair natural orbital (PNO) threshold toward the complete PNO space (CPS) limit for the DLPNO methods.^99^ The PNO threshold (T_CutPNO_) controls the number of PNOs included for each electron pair based on the occupation number.^70^ The coupled-cluster equations are then solved for the compacted virtual space for each electron pair spanned by the PNOs above the threshold. To the best of our knowledge, the CPS extrapolation scheme has not been explored for atmospheric molecular clusters.

Recently, atmospheric cluster studies have expanded to studying much larger clusters (20–30 monomers);^100–105^ for these sizes, while possible, the NormalPNO DLPNO–CCSD­(T_0_)/aug-cc-pVTZ method’s memory per core requirements result in very inefficient usage of computational resources. Therefore, lower basis sets and PNO settings are needed to perform these computations efficiently. However, employing extrapolation schemes with lower settings calculations may help reduce the loss of accuracy compared to the usually employed NormalPNO DLPNO–CCSD­(T_0_)/aug-cc-pVTZ method.

Alternatively, other local coupled cluster methods may be used. The MRCC team’s local natural orbital (LNO) methods^106–111^ offer similar performance and accuracy. The LNO method shares the core idea of DLPNO, by solving CCSD­(T) equations in localized ”domains” where electron correlation is strongest but one of the key differences is that it does not use PNOs. Instead, it employs a (not-pair-specific) LNO basis, which is constructed from a MP2 density matrix of the extended domain around a central localized orbital. Similar to the DLPNO method’s LoosePNO, NormalPNO, and TightPNO settings, the LNO method comes with predefined approximation settings (VeryLoose, Loose, Normal, Tight, VeryTight, VeryVeryTight) that set the truncation and residual thresholds used throughout the method, ensuring they converge toward the canonical limit. These predefined approximation settings are extrapolatable to the local free approximation limit (LAF).^112^ To the best of our knowledge, the LNO methods have not been explored for atmospheric molecular clusters.

In this study, we benchmark local coupled cluster methods using different basis sets and test extrapolation schemes to the CBS, LAF, and CPS limits. The extrapolation procedures are tested against the binding energies of high-level CCSD­(F12*)­(T+)/cc-pVTZ-F12 reference calculations. We applied an extended test set compared to previous work, comprising RI-MP2/aug-cc-pVTZ equilibrium structures for all possible monomers and dimers (except double base structures) of SA, FA, NA, MSA, W, AM, MA, DMA, TMA, and EDA. In addition, we also include all unique conformers above a root-mean-squared deviation (RMSD) threshold of 0.38 Å, leading to a test set of a total of 218 cluster systems.

## 2 Methodology

### 2.1 Computational Details

Configurational sampling was performed using CREST 2.12 ^113,114^ in noncovalent interaction (NCI) mode, employing GFN1-xTB.^115^ Optimization and numeric frequencies at the RI-MP2/aug-cc-pVTZ level of theory were performed using ORCA 5.0.4.^116–118^ The DLPNO–CCSD­(T_0_)^70–75^ calculations were performed using ORCA 6.0.0.^116–118^

The CCSD­(F12*)­(T+),^119–121^ CCSD­(T),^122–124^ and LNO–CCSD­(T)^106–112^ calculations were performed using MRCC (august 28, 2023 version).^125,126^

The basis employed for each method is given in Table 1. AutoAux^127^ was used for the double-ζ basis set calculations in ORCA. aug′ is the augmented basis sets without the extra augmentation of hydrogen (i.e, cc-pV*X*Z for hydrogen, aug-cc-pV*X*Z for the other atoms). Same-sized, matching auxiliary RI-JK^128^ and RI^129,130^ basis sets were employed for the MRCC calculations. Similarly, the matching auxiliary/C and/JK auxiliary basis sets were employed for the ORCA calculations, except for the double-ζ calculations, where AutoAux was used, as ORCA does not have a matching double-sized auxiliary basis set.

**Table 1:** Basis Sets Used for the Different Methods

Method	Basis Sets	Cardinal number
CCSD(T)	aug-cc-pV*X*Z	*X* = D, T, Q
CCSD(F12*)(T+)	cc-pV*X*Z-F12	*X* = D, T, Q
LNO–CCSD(T)	aug-cc-pV*X*Z	
	aug′-cc-pV*X*Z	*X* = D, T, Q
	cc-pV*X*Z	
DLPNO–CCSD(T_0_)	aug-cc-pV*X*Z	
	aug′-cc-pV*X*Z	*X* = D, T, Q
	cc-pV*X*Z	AutoAux for D

### 2.2 Constructing the Test Set

We studied all dimer cluster combinations consisting of the SA, FA, NA, MSA, W, AM, MA, DMA, TMA, and EDA molecules. We excluded the (base)_2_ clusters as these are very unstable under atmospheric conditions. The initial dimer structures were taken from the study of Schmitz and Elm.^77^ All the structures were subsequently optimized at the GFN1-xTB level of theory and the lowest electronic energy conformer was used as the starting point for an NCI mode CREST simulation. All nonunique conformers from CREST were removed using a modified version of the ArbAlign program,^131^ based on a root-mean-squared deviation threshold of 0.38 Å as suggested by Kildgaard et al.^132,133^ The remaining unique structures were optimized at the RI-MP2/aug-cc-pVTZ level of theory. All conformers with real vibrational frequencies were kept for the benchmark. This led to a test set of a total of 218 cluster configurations. The number of individual configurations for each cluster system is presented in .

### 2.3 ACDC Simulations

The birth–death equations were generated via the Atmospheric Cluster Dynamics Code (ACDC)^67,134^ for the (SA)_1–4_(AM/DMA)_1–4_ structures by Kubečka et al.^25^ The (SA)_5_(base)_4_ (SA)_4_(base)_5_ were set as the outgrowing clusters. All single acid clusters were counted toward the total acid concentration. Size-dependent coagulation sinks were activated using values of cs_exp = −1.6 and cs_ref = 1 × 10^–3^ s^–1^. The coagulation sink parameters were chosen to match typical values for the boundary layer.^135^

The Jammy Key framework^136^ was used to automate the workflow, data processing, and setup of the ACDC simulations.

### 2.4 Extrapolation Schemes

For the basis set extrapolations, we use the two-point extrapolation scheme with coefficients suggested by Neese and Valeev.^137^ Here the SCF energy can be extrapolated as^93^

3 $$E_{\mathrm{CBS}}^{\mathrm{SCF}}=\frac{E_{X}^{\mathrm{SCF}}S\left(X+1\right)-E_{X+1}^{\mathrm{SCF}}S\left(X\right)}{S\left(X+1\right)-S\left(X\right)}$$

where *X* is the cardinal number of the smallest basis set and *S* is an exponential function given as

4 $$S\left(Q\right)=\exp\left(-\alpha \sqrt{Q}\right)$$

where *Q* is the cardinal number given as input and α is an extrapolation coefficient.

The correlation energy is extrapolated as^85^

5 $$E_{\mathrm{CBS}}^{\mathrm{corr}}=\frac{X^{\beta }E_{X}^{\mathrm{corr}}-{\left(X+1\right)}^{\beta }E_{X+1}^{\mathrm{corr}}}{X^{\beta }-{\left(X+1\right)}^{\beta }}$$

where β is another extrapolation coefficient.

The extrapolation coefficients are given in Table 2. It should be noted that for the aug′ basis sets, as specific coefficients have not been fitted, we used the corresponding extrapolation coefficient from the fully augmented basis sets. This decision was based on the insensitivity between the extrapolation coefficients of the cc-pV*X*Z and aug-cc-pV*X*Z basis sets, and that the aug′-cc-pV*X*Z basis set is most similar to the aug-cc-pV*X*Z basis set. Initial testing also showed it did not lead to any obvious outliers.

**Table 2:** Extrapolation Coefficients Suggested by Neese and Valeev^137^ ^,^ ^a^

Basis	α(2, 3)	β(2, 3)	α(3, 4)	β(3, 4)
cc-pV*X*Z	4.42	2.46	5.46	3.05
aug′-cc-pV*X*Z	4.30	2.51	5.79	3.05
aug-cc-pV*X*Z	4.30	2.51	5.79	3.05

a Note that the reduced basis set uses the same coefficients as the fully augmented basis set. The numbers in the parentheses of the coefficients are the cardinal numbers for the extrapolation.

The CPS and LAF extrapolation follow the same form with the same extrapolation coefficient of *F* = 1.5,

6 $$E_{\mathrm{CPS}/\mathrm{LAF}}=E_{\mathrm{LOW}}+F\left(E_{\mathrm{HIGH}}-E_{\mathrm{LOW}}\right)$$

where LOW refers to the lower-level setting and HIGH to the higher-level setting. The LNO methods come with predefined locality settings, where two successive settings can be used to extrapolate to the LAF limit. For the CPS extrapolation, the PNO cutoff was 1 order of magnitude higher for the high-level, while the other parameters (*T*_CutPairs_ = 10^–4^ and *T*_CutMKN_ = 10^–3^) were kept fixed according to the NormalPNO settings.^138^ An example of a lower-level and higher-level setting would be NormalPNO at 3.33 × 10^–7^ and NormalPNO with 3.33 × 10^–8^, respectively, for the DLPNO method. For the LNO method, it could be from the “Normal” and “Tight” locality settings, respectively.

We will denote a basis set extrapolation using cardinal numbers *X* and *X* + 1 as CBS­(*X*,*X* + 1). For the augmented basis set, we will use “aug-” or “aug′-” in front of the cardinal numbers. A DLPNO calculation with the PNO threshold set at 3.33 × 10^–T^ will be denoted PNO­(*T*). Extrapolations of PNO settings will use the CPS­(*T*,*T* + 1) notation. The LNO locality setting will follow the naming convention of the MRCC manual. Extrapolation of the settings will be denoted LAF­(LOW,HIGH), where the setting name is shortened until the first capital letter. (i.e., vLoose and Loose is denoted LAF­(vL,L)).

Five settings were probed for the DLPNO methods: NormalPNO with the PNO threshold set from 2 orders of magnitude lower to 2 orders of magnitude higher. Six settings for the LNO methods: vvLoose to vvTight. The basis sets tested were cc-pV*X*Z, aug′-cc-pV*X*Z (no augmentation on hydrogen), and aug-cc-pV*X*Z (*X* = D,T,Q).

### 2.5 Binding Energies

The studied property is the electronic binding energy, which is defined as the electronic energy required to bring the monomers together from infinitely far apart to form the cluster,

7 $$\Delta E^{\mathrm{cluster}}=E^{\mathrm{cluster}}-\underset{n}{\sum }E_{n}^{\mathrm{monomer}}$$

where *E*_cluster_ is the electronic energy of the dimer, and *E*_monomer_ is the electronic energy of the monomer making up the dimer. The monomer and dimer electronic energies are always computed at the same level of theory.

## 3 Results and Discussion

### 3.1 Reference Methods

A suitable reference is needed to benchmark the local coupled cluster methods. We chose the F12 methods as they have been shown to give results close to the CBS limit for binding energies of atmospheric molecular clusters.^77^ We used the (T+) triple treatment, which is a size-consistent version of the (T*) treatment, because it exhibits smaller basis set error for binding energies.^119^ To further validate the convergence of the CCSD­(F12*)­(T+) methods, we calculated the binding electronic energies from cc-pVDZ-F12 to cc-pVQZ-F12 of the SA–W, SA–AM, FA–W, and FA–W dimer clusters, as these are small enough for the cc-pVQZ-F12 calculation and span the acid–water, acid–base, organic–water, and organic–base interactions, respectively. We also compared these to the canonical CCSD­(T)/CBS­(aug-2,aug-3), CCSD­(T)/CBS­(aug-3,aug-4), and CCSD­(T)/CBS­(2,3,4) binding energies.

The calculated binding energies can be seen in Table 3. All the F12 calculations give binding electronic energies within 0.09 kcal/mol of each other, with the largest difference being between cc-pVDZ-F12 and cc-pVQZ-F12 for the FA–AM dimer and cc-pVDZ-F12 and cc-pVTZ-F12 for the SA–W dimer. This indicates that binding energies are already well converged at the F12 level of theory when using the cc-pVDZ-F12 basis set. Similarly, if we compare the F12 values to the extrapolated canonical CCSD­(T) results, we get within 0.1 kcal/mol between cc-pVQZ-F12 and CBS­(aug-3,aug-4). The CBS­(aug-2,aug-3) energies diverge a bit more with a deviation of up to 0.5 kcal/mol compared to the cc-pVQZ-F12 results. This suggests that we might need to include quadruple-size basis sets in the extrapolation procedure to get well-converged energies. Finally, the CBS­(2,3,4) 4–5 inverse polynomial extrapolation^85^ used by the Shields’ group^36,95,96,96–98^ also performs similarly to the F12 results with the largest deviation, to the cc-pVQZ-F12 result, being 0.2 kcal/mol for the FA–AM dimer.

**Table 3:** Binding Electronic Energies of the Small Dimers for the CCSD­(F12*)­(T+) and CCSD­(T) Calculations as a Function of the Basis Set Size and Extrapolation Scheme^a^

Dimer	cc-pVDZ-F12	cc-pVTZ-F12	cc-pVQZ-F12	CBS(aug-2,aug-3)	CBS(aug-3,aug-4)	CBS(2,3,4)
SA–AM	–16.314	–16.344	–16.290	–16.704	–16.350	–16.158
SA–W	–12.698	–12.784	–12.735	–13.245	–12.826	–12.683
FA–AM	–11.622	–11.588	–11.534	–11.814	–11.558	–11.317
FA–W	–10.402	–10.438	–10.382	–10.718	–10.445	–10.225

a The CBS­(2,3,4) is the 4–5 inverse polynomial extrapolation^85^ used by the Shields’ group,^36,95,96,96–98^ where we extrapolated the total electronic energy according to the formula given in the SI of Longsworth et al.^96^ Units are in kcal/mol.

The CCSD­(F12*)­(T+)/cc-pVQZ-F12 binding energies would have been ideal as a reference, but these become too computationally expensive for the larger dimers in our test set. Therefore, we chose the CCSD­(F12*)­(T+)/cc-pVTZ-F12 binding energies as the reference values, as these are feasible for all the dimer clusters and well-converged. However, this also implies that we will have to accept an intrinsic error on the order of 0.1 kcal/mol, which is still a much lower error than current cluster formation studies.

### 3.2 Dimer Clusters

We calculated the binding energies of the entire test set for all combinations of basis sets, extrapolations of basis sets, locality settings, and extrapolations of locality settings for the DLPNO–CCSD­(T_0_) and LNO–CCSD­(T) methods. Including all possible ways of extrapolating yields 300 different ways of calculating the electronic binding energy for each system. Due to computational limits, not all systems have been converged for the PNO(9) calculations.

#### 3.2.1 aug-cc-pV*X*Z

We start by comparing the results from the largest studied basis sets, aug-cc-pV*X*Z. The results for the DLPNO method are shown in Figure 1 and the results for the LNO method in Figure 2. From the DLPNO results, it is clear that the aug-cc-pVDZ basis set is too small (first row of Figure 1). We observe a mean absolute error (MAE) of roughly 0.7 kcal/mol and a max absolute error of roughly 2.4 kcal/mol for all PNO settings and extrapolations. The insensitivity to the PNO threshold confirms that the basis set error is the dominating error. The aug-cc-pVTZ calculations halve (≈ 0.3 kcal/mol) the MAE compared to the aug-cc-pVDZ results. Furthermore, the CPS­(6,7) extrapolation is needed before the basis set error dominates, as the max absolute error is the same at this setting/extrapolation and higher. For the usually employed PNO(7) DLPNO–CCSD­(T_0_)/aug-cc-pVTZ level of theory, we have a low MAE of 0.28 kcal/mol, however, the max absolute error is still quite substantial at 1.1 kcal/mol. These errors are consistent with the previous results by Schmitz and Elm.^77^ The large max error is mainly caused by the tail of underbinding (to the right) clusters comprising the (MSA)_1_(EDA)_1_, (MSA)_1_(MA)_1_, and (SA)_1_(MA)_1_ clusters. The max error could be reduced to 0.82 kcal/mol by performing the PNO(6) calculation and extrapolation between it and the PNO(7) setting.

**Figure 1:**
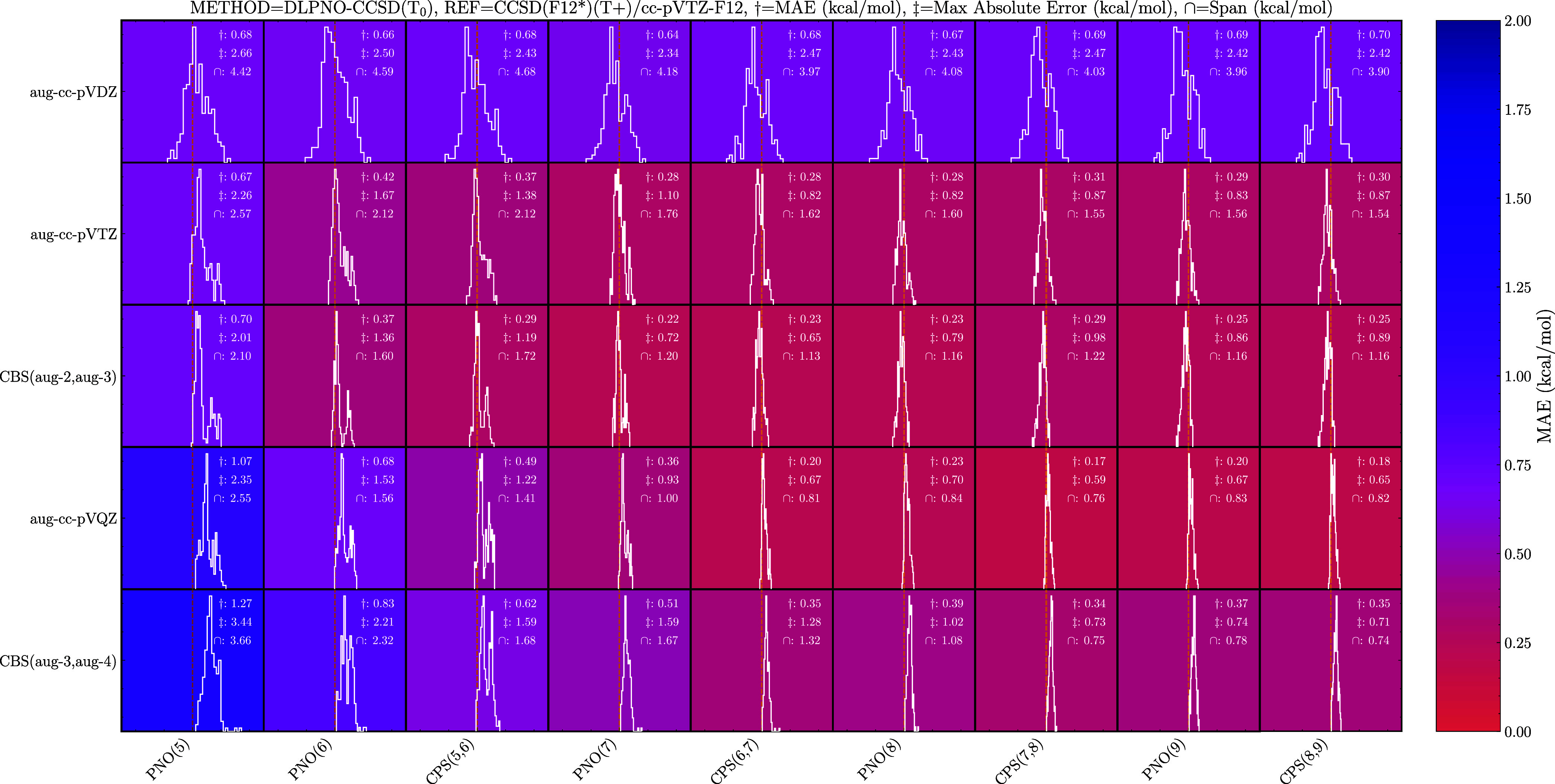
Binding energy errors of the DLPNO method using the augmented basis sets compared to the CCSD­(F12*)­(T+)/cc-pVTZ-F12 level of theory. The PNO­() labels indicate calculations performed at NormalPNO settings with the PNO thresholds set using the number as the exponent. CPS­(,) and CBS­(,) denote complete PNO space and complete basis set extrapolations, respectively. Each box spans 10 kcal/mol. Negative values (left) indicate overbinding relative to the reference method.

**Figure 2:**
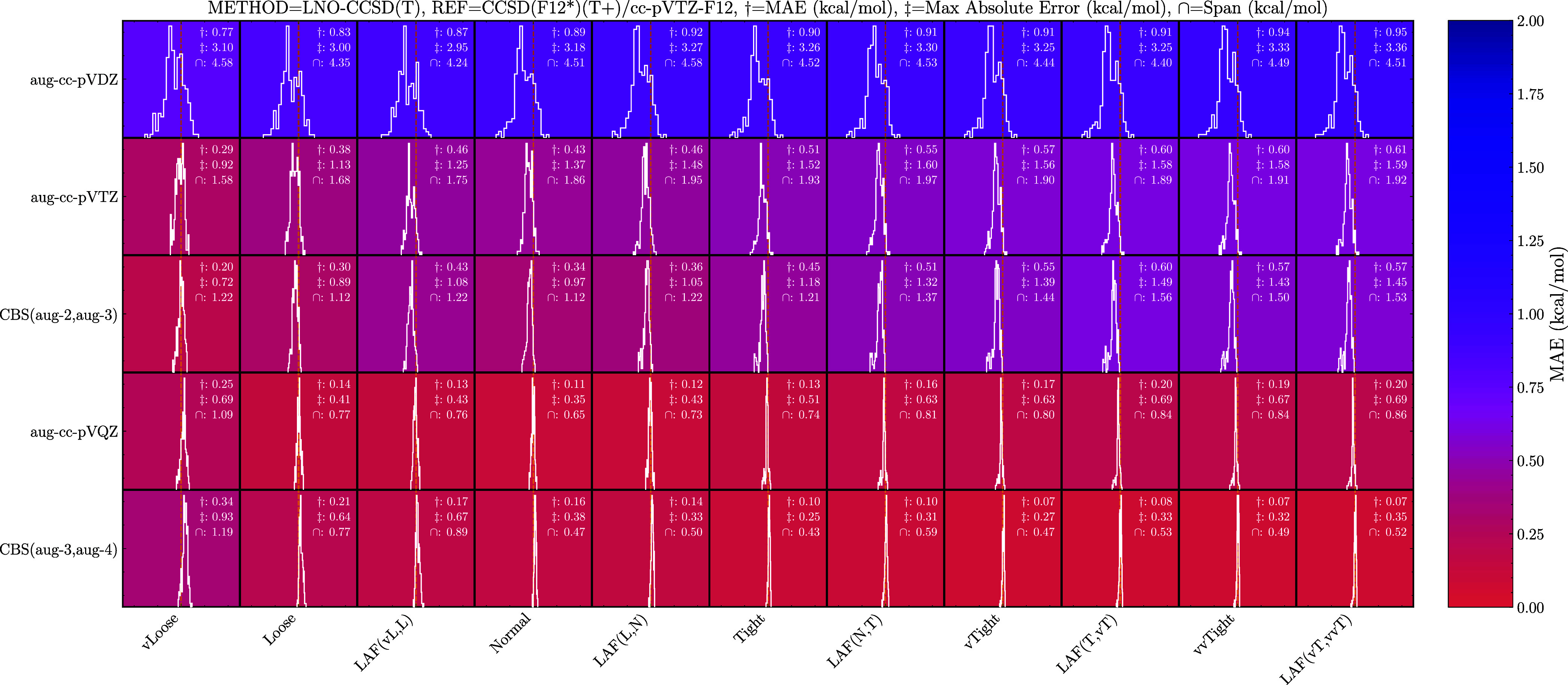
Binding energy errors of the LNO method using the augmented basis sets compared to the CCSD­(F12*)­(T+)/cc-pVTZ-F12 level of theory. LAF­(,) and CBS­(,) denote local approximation free and complete basis set extrapolations, respectively. Each box spans 10 kcal/mol. Negative values (left) indicate overbinding relative to the reference method.

Extrapolating between the double- and triple-ζ basis sets always reduces the MAE, except for the PNO(5) setting. Following along the row, the max absolute error does not always get lower when extrapolating; this happens for the CPS­(7,8) extrapolation and above. This is likely due to the loss of cancellation of errors for the CPS­(7,8) extrapolation compared to the PNO(7) setting. This also shows that routine cluster formation studies employing the normal PNO(7) setting with an aug-cc-pVTZ basis set could obtain increased accuracy by performing CBS­(aug-2,aug-3) extrapolation.

Using the aug-cc-pVQZ basis set reduces the span of errors. However, there is an increase in the MAE for the PNO(7) setting and below. This is due to a general trend where a larger basis set causes all the errors to shift toward overbinding (to the left), whereas the locality error shifts it toward underbinding (to the right). For the CPS­(6,7) extrapolation and higher, the reduced error in the locality approximation reduces the spread and hence the MAE. Extrapolating between the triple and quadruple basis sets again does not necessarily decrease the MAE, as the previous basis set sizes allowed for the basis set error to partly cancel with the locality error, allowing the error distribution to be centered around zero. Following along the row for the CBS­(aug-3,aug-4) results, the error distributions start around zero, with the PNO settings controlling the span. The remaining local error is unlikely to be due to the (T_0_) approximation as Schmitz and Elm^77^ found minimal change in the error distribution using the (T_1_) approach compared to the CCSD­(T)-F12/CBS reference. A way to lower the local error would be to add the F12 correction. Schmitz and Elm^77^ showed this can significantly lower the error, but it comes with a memory requirement that would make it unsuitable for larger molecular clusters.

In general, we observe that the commonly applied DLPNO–CCSD­(T_0_)/aug-cc-pVTZ results rely on the cancellation of errors between the basis set error and the locality error to give an error distribution centered around zero. For the fully augmented basis set, a DLPNO–CCSD­(T_0_)/CBS­(aug-2,aug-3) energy calculation using the PNO(7) settings would be an ideal compromise between accuracy and computational cost.

For the LNO method, the small aug-cc-pVDZ basis set still yields large basis set errors with MAEs of roughly 0.9 kcal/mol and max absolute errors of up to 3.36 kcal/mol. Tightening the locality setting does not significantly change the MAE, as the basis set error dominates. It only shifts the error distribution toward overbinding (left). Similar to DLPNO, increasing the basis set size to aug-cc-pVTZ roughly halves the MAE and max absolute error in all cases. When tightening the locality setting, we see a shift in the error distribution toward overbinding (left) causing it to deviate from zero. Doing an extrapolation between the basis sets, does not significantly change the MAE, but it reduces the span of errors. For instance, going from Normal LNO–CCSD­(T)/aug-cc-pVTZ to Normal LNO–CCSD­(T)/CBS­(aug-2,aug-3) reduces the error span from 1.86 to 1.12 kcal/mol. Similarly to the DLPNO results, tightening the locality setting does not decrease the MAE as the error distribution is already centered around zero at the vLoose setting. For the tighter locality settings, the max absolute error increases due to a tail of larger errors from the (MSA)_1_(SA)_1_, (MSA)_2_, and (SA)_2_ dimer clusters. Calculating the energies at the aug-cc-pVQZ level massively reduces the MAE by a factor of 3 compared to the CBS­(aug-2, aug-3) results and decreases the max absolute error by more than a factor of 2 for all locality settings except vLoose. This massive decrease in error is due to the distribution exhibiting less spread but also a near-perfect cancellation of errors where the basis set error shifts toward underbinding (right) but the locality error shifts toward overbinding (left). This combination results in a narrow error distribution centered on zero for the Normal LNO–CCSD­(T)/aug-cc-pVQZ level of theory. This is also the reason the results do not necessarily improve when performing the CBS­(aug-3,aug-4) extrapolation, since we end up in the same scenario as the DLPNO method, where the error distribution roughly starts at zero, with the tightness of the locality setting controlling the width.

In general, we find that, at comparable settings to the usually employed NormalPNO DLPNO–CCSD­(T_0_)/aug-cc-pVTZ calculation, DLPNO benefits from excellent cancellation of errors. This ideal cancellation only happens for the LNO method with the larger aug-cc-pVQZ basis set. However, the error distribution is much narrower for the LNO methods at the larger basis set sizes compared to the DLPNO method, suggesting the locality error is lower for the LNO methods. Furthermore, we find the LNO methods to be much more computationally efficient (will be discussed in greater detail in sections 3.3 and 3.5) compared to similar settings for DLPNO. This means that more accurate LNO calculations are readily achievable on larger systems. Since the goal is to achieve the lowest error possible on all system sizes, we will, from now on, focus on the LNO methods; however, we have added the equivalent DLPNO benchmarks for the cc-pV*X*Z and aug′-cc-pV*X*Z basis sets in the .

#### 3.2.2 aug′-cc-pV*X*Z

Due to the noncovalent bonded nature of atmospheric clusters, diffuse functions should, in principle, be added to the basis functions to better describe the interactions. The problem is that these functions can substantially increase the computational cost. Furthermore, they might cause SCF convergence problems due to linear dependencies in the basis.^104^ A compromise would be to only augment all non-hydrogen atoms with diffuse functions. The Dunning basis sets following this augmentation philosophy are denoted the aug′-cc-pV*X*Z basis sets, and the results for the LNO method are shown in Figure 3 and the DLPNO method in .

**Figure 3:**
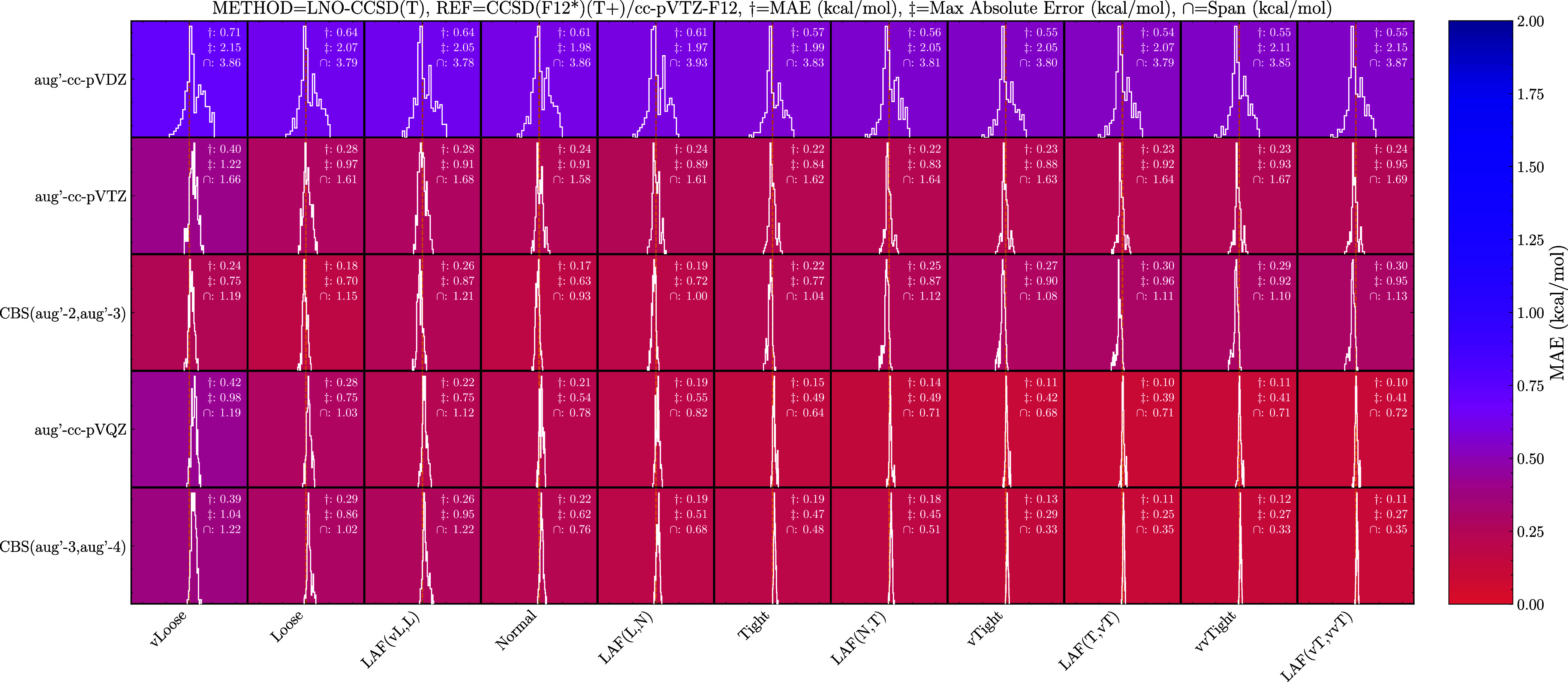
Binding energy errors of the LNO method using the reduced augmented basis sets compared to the CCSD­(F12*)­(T+)/cc-pVTZ-F12 level of theory. LAF­(,) and CBS­(,) denote local approximation free and complete basis set extrapolations, respectively. Each box spans 10 kcal/mol. Negative values (left) indicate overbinding relative to the reference method.

The aug′-cc-pVDZ basis set does still not perform well, with a MAE of 0.54–0.71 kcal/mol and max absolute errors of 1.97–2.15 kcal/mol. Interestingly, at the aug′-cc-pVTZ basis set size, the basis set error makes the error distribution centered at zero for the Normal setting. This yields similar errors to the aug-cc-pVTZ DLPNO method but for a smaller basis set. However, tightening the locality setting does not significantly increase the accuracy, as the basis set error still dominates. For the vTight setting and higher, the max absolute error increases because the error distribution shifts away from being centered at zero. Similarly, the CBS­(aug′-2,aug′-3) extrapolation does not perform much better due to the same reason, however, the error span does improve, going from roughly 1.5 kcal/mol to roughly 1.1 kcal/mol compared to the aug′-cc-pVTZ results. Increasing the basis set size to aug′-cc-pVQZ does not decrease the MAE for the Tight setting and lower. The max absolute error also slightly decreases at the LAF­(vL,L) extrapolation and higher. Similarly, performing the CBS­(aug′3-aug′-4) extrapolation does not significantly change the accuracy and, for several cases, actually decreases the accuracy a bit. However, the extrapolation does benefit from tighter locality settings as the three error measures can be halved by going from Normal to vTight. While the error measures are higher than the equivalent locality setting for the augmented basis set, the error distribution is still narrow, and the slight increase in error might be worth the gain in computational efficiency.

#### 3.2.3 cc-pV*X*Z

While the nonaugmented basis might lack the extra diffuse functions, the extrapolations might make up for it, and being able to use them would result in a massive increase in computational speed compared to the augmented basis sets. The results for the LNO methods are shown in Figure 4 and the DLPNO method in . The smaller cc-pVDZ and cc-pVTZ basis sets have the largest max absolute errors of up to 5.52 and 2.21 kcal/mol, respectively. While the cc-pVTZ and CBS­(2,3) cases have reasonable MAEs down to 0.62 and 0.31 kcal/mol, respectively, the large max absolute error still makes them less than ideal, yet still shows an improvement over the original DLPNO method. Increasing the basis set size to cc-pVQZ decreases the MAE and max absolute error because the error distribution gets centered around zero; however, the error span largely stays the same with and without extrapolation at this basis set size. Furthermore, for all the basis sets at or below this size, there is not a substantial change in accuracy when increasing from the Normal locality setting and above, indicating the basis set error is quite substantial for the nonaugmented basis sets. Interestingly, while the previous basis sets sizes and extrapolation did not perform as well as the equivalent (reduced) augmented basis set. The CBS­(3,4) extrapolation performs excellently. For instance, the Normal CBS­(3,4) extrapolation has a MAE of 0.11 kcal/mol, a max absolute error of 0.37 kcal/mol, and an error span of 0.61 kcal/mol. It performs as well as the equivalent augmented basis set and setting (MAE of 0.16 kcal/mol, max ABS of 0.38 kcal/mol, error span of 0.47 kcal/mol). Furthermore, it outperforms the equivalent reduced augmented basis set and setting with nearly half the MAE and max absolute error and a similar error span.

**Figure 4:**
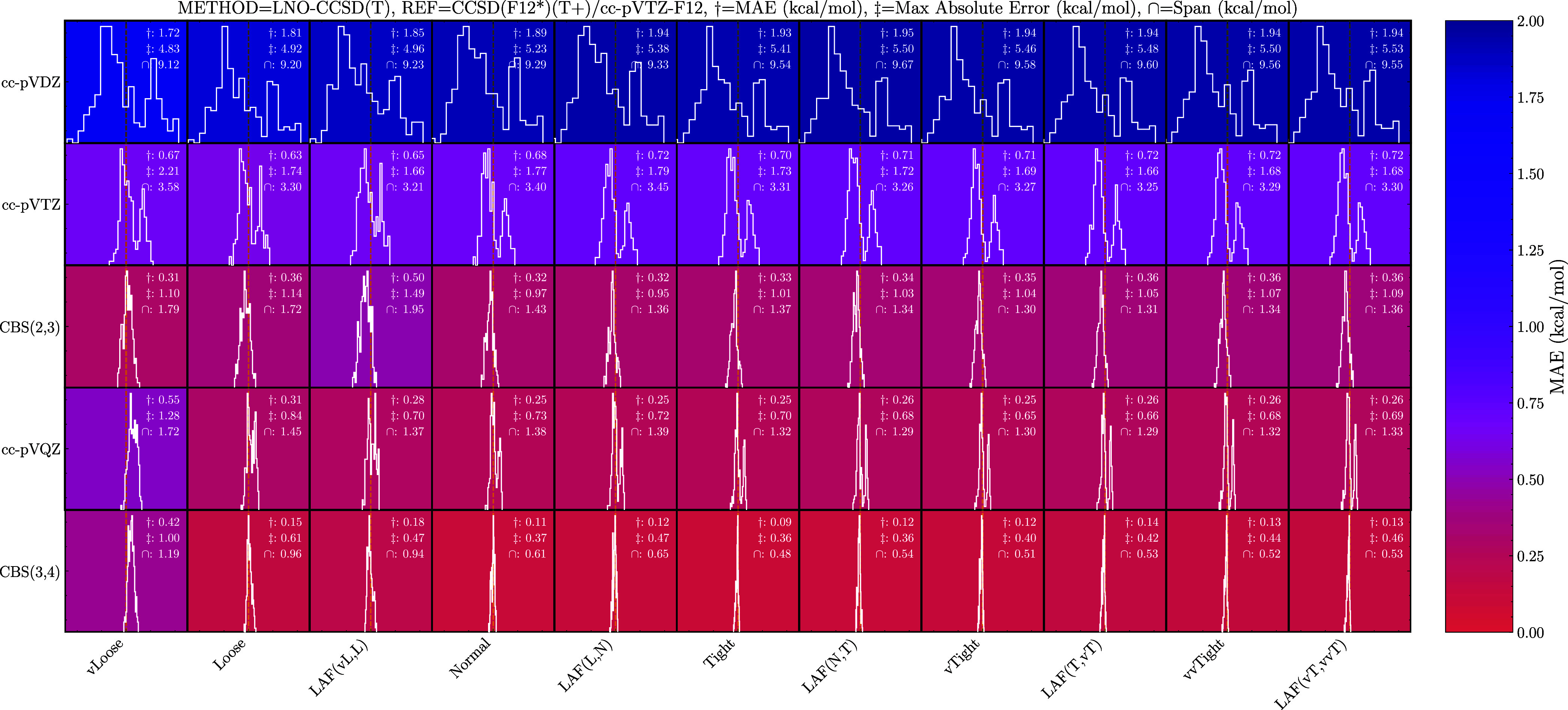
Binding energy errors of the LNO method using the nonaugmented basis sets compared to the CCSD­(F12*)­(T+)/cc-pVTZ-F12 level of theory. LAF­(,) and CBS­(,) denote local approximation free and complete basis set extrapolations, respectively. Each box spans 10 kcal/mol. Negative values (left) indicate overbinding relative to the reference method.

This indicates that the Normal LNO–CCSD­(T)/CBS­(3,4) level of theory might be a sweet spot between accuracy and computational cost for calculating the binding energies of (smaller) atmospheric molecular clusters. However, as this might not hold for larger clusters (will be discussed in Section 3.4), we still recommend applying LNO–CCSD­(T)/CBS­(aug-3,aug-4) with at least the Normal setting for the usually studied cluster sizes of 2–8 monomers, as these should be readily attainable.

### 3.3 Timing Trends of the Different Methods

Figure 5 shows the walltime for a LNO–CCSD­(T) single-point calculation of (SA)_1_(DMA)_1_ using the given settings relative to the usually employed NormalPNO DLPNO–CCSD­(T_0_)/aug-cc-pVTZ using 10 cores. It should be noted that these relative timings will change for larger system sizes, and this is primarily for a quick estimate.

**Figure 5:**
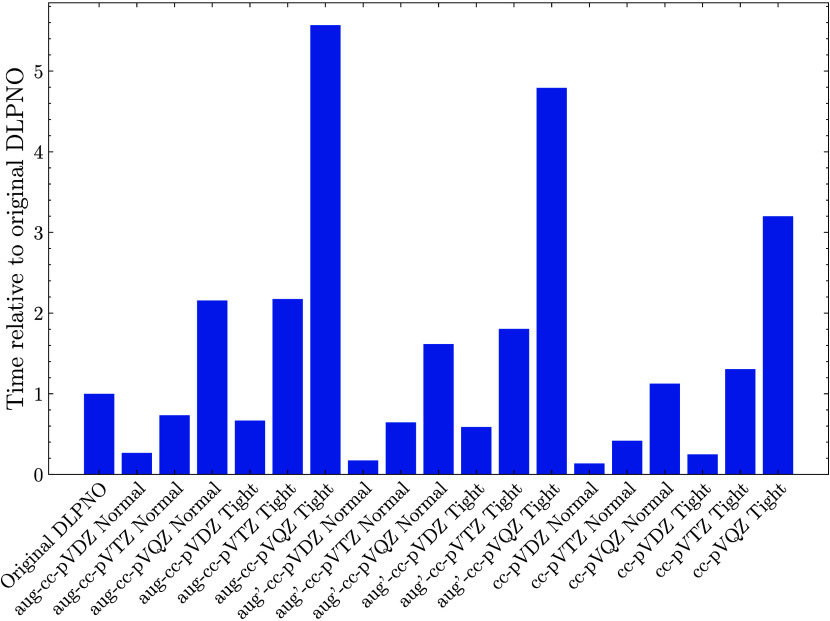
Walltime for a LNO–CCSD­(T) single-point calculation of (SA)_1_(DMA)_1_ using the given settings relative to the usually employed NormalPNO DLPNO–CCSD­(T_0_)/aug-cc-pVTZ using 10 cores. The calculations were performed using 10 parallel MPI processes in ORCA or 10 OpenMP threads in MRCC, both on an Intel Xeon Platinum 8358 CPU using a dedicated local disk.

Increasing from the Normal to Tight setting increases the time by a factor of 2–3, but it does not substantially change the accuracy for the dimer test set. A similar trend is observed for augmented versus the nonaugmented basis sets with a factor of 2 difference. Reducing the basis set from the augmented to the reduced augmented aug′ basis does not significantly change the walltime, but comes with a slight increase in error for the dimers. However, we find that for much larger clusters (see Section 3.5) the timing difference is much larger and the fully augmented basis set calculations are not possible, but the reduced augmented are.

Comparing to the original DLPNO method, it is possible to perform the Normal CBS­(aug-2,aug-3), Normal CBS­(aug′-2,aug′-3), or Normal CBS­(2,3) calculation for the same amount of time. Likewise, using a bit more time, it is possible to perform the Normal CBS­(3,4) calculation, which showed excellent accuracy in the dimer benchmark.

### 3.4 The Effects on Simulated New Particle Formation Rates

As concluded in Section 3.2, an extrapolation scheme is needed to reduce the basis set error and obtain accurate binding energy results. For the most part, the benchmark indicates the error measures are fairly insensitive to the tightness of the locality setting as long as they are at or above the Normal setting. However, the benchmark is limited to the dimer systems as these are the only ones small enough to obtain a high-level reference for. The goal is to simulate the new particle formation rate with cluster sizes much larger than the dimers. To probe this, we test the sensitivity of the different extrapolations’ effect on the simulated new particle formation rates of the (SA)_1–4_(DMA)_1–4_ and (SA)_1–4_(AM)_1–4_ systems sampled by Kubečka et al.^25^ We keep the locality setting at Normal or Tight, and extrapolation of them due to the aforementioned lack of sensitivity.

Figure 6 shows the simulated new particle formation rates of the (SA)_1–4_(DMA)_1–4_ cluster system using single points at all possible (2,3) and (3,4) extrapolations along with the Normal and Tight and the LAF­(N,T) extrapolation. The temperature was set to 278.15 K with an SA concentration of either 10^7^ molecules cm^–3^ (denoted high) or 10^6^ molecules cm^–3^ (denoted low) and 10 ppt of DMA. The original DLPNO bar is the NormalPNO DLPNO–CCSD­(T_0_)/aug-cc-pVTZ level.

**Figure 6:**
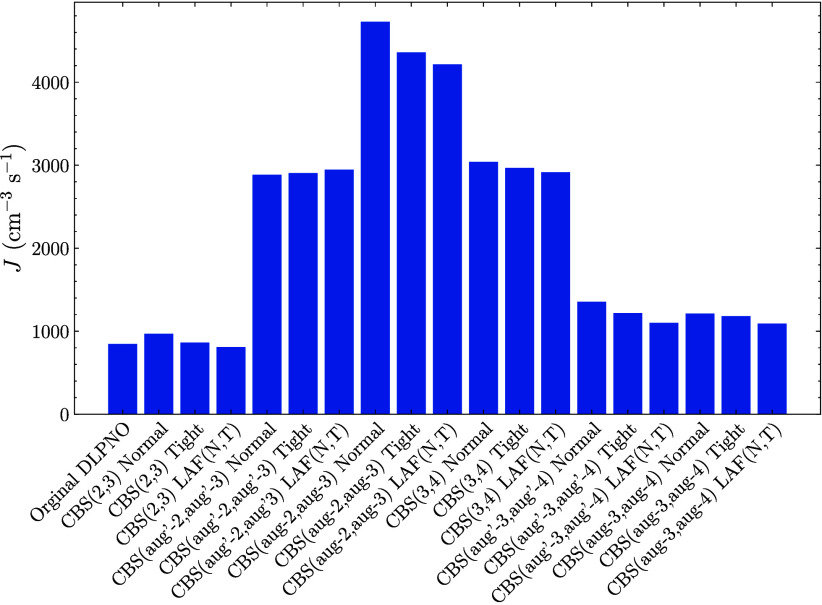
New particle formation rate as calculated by ACDC for the (SA)_1–4_(DMA)_1–4_ system with the single point at the given LNO–CCSD­(T) level given by the labels. The thermal contribution is calculated at the ωB97X-D/6-31++G­(d,p) level of theory. The temperature was set to 278.15 K, the SA concentration at 10^7^ molecules cm^–3^, and the DMA concentration at 10 ppt.

#### 3.4.1 High SA Concentration

For simulations using high SA concentrations, even with the exponential dependence on the free energy, all the different binding energy schemes yield new particle formation rates within a factor of 5 of each other. Hence, simulated NPF rates are relatively insensitive to the choice of high-level methods for calculating the binding energies. The rates are also relatively insensitive to the locality setting at this concentration, with the largest relative change being a drop by 369 cm^–3^ s^–1^ for the Normal to Tight setting at the CBS­(aug-2,aug-3) level. It should be noted that this is also the largest total rate.

The behavior of the rates can be explained by studying the Gibbs free energies under the given conditions (see ). We observe that for all levels of theory, the initial dimer formation constitutes the highest nucleation barrier, and the rate directly follows its free energy. For instance, the DLPNO, CBS­(2,3), CBS­(aug′-3,aug′-4) and CBS­(aug-3,aug-4) single-points all predict the dimer free energy under given conditions to be around 2.5–2.8 kcal/mol, whereas the CBS­(aug′-2,aug-’3) and CBS­(3,4) levels predict it to be around 2.1–2.2 kcal/mol, and the CBS­(aug-2,aug-3) level around 1.9–2.0 kcal/mol.

From the high-concentration simulations, we can conclude that for systems or concentrations where the initial dimer is the rate-determining step, the results are somewhat insensitive to the level of theory as long as they predict the dimer free energy similarly. For instance, the NormalPNO DLPNO–CCSD­(T_0_)/aug-cc-pVTZ level and the CBS­(2,3) Tight level both predict a NPF rate of roughly 850 cm^–3^ s^–1^ due to identical free energy of the dimer cluster, despite the (SA)_4_(DMA)_4_ cluster being 3.7 kcal/mol lower in free energy for the DLPNO method.

#### 3.4.2 Low SA Concentration

The NPF rate is also dependent on the precursor concentrations. Changing the concentration will change the rate-determining step. Figure 7 shows the simulated NPF rates at a SA concentration of 10^6^ cm^–3^. Decreasing the concentrations expands the rate-determining clusters to include the (SA)_2_(DMA)_1_ cluster. Furthermore, the (SA)_2_(DMA)_2_ cluster also has positive free energy at the given conditions for the CBS­(3,4) Tight setting and above. The high-dimensional parameter space makes it difficult to directly couple the overall NPF rate to specific cluster free energies, but some general trends can be observed. Overall, the NPF rates vary more compared to the high-concentration scenario because the important cluster free energies are no longer predicted similarly between the methods. For instance, the rates, compared to the high SA concentration, decrease substantially with increasing tightness of the locality settings. The DLPNO–CCSD­(T_0_)/aug-cc-pVTZ rate of 1.57 cm^–3^ s^–1^ is also substantially higher than the CBS­(aug-3,aug-4) results (0.13–1.57 cm^–3^ s^–1^). This indicates that for systems where the rate is low and several clusters in the simulated system are rate-determining, the different methods can no longer be expected to yield similar results. However, as in the high SA concentration simulation, the differences between the CBS­(aug′-3,aug′-4) and CBS­(aug-3,aug-4) results for the same locality settings are low (below 0.03 cm^–3^ s^–1^).

**Figure 7:**
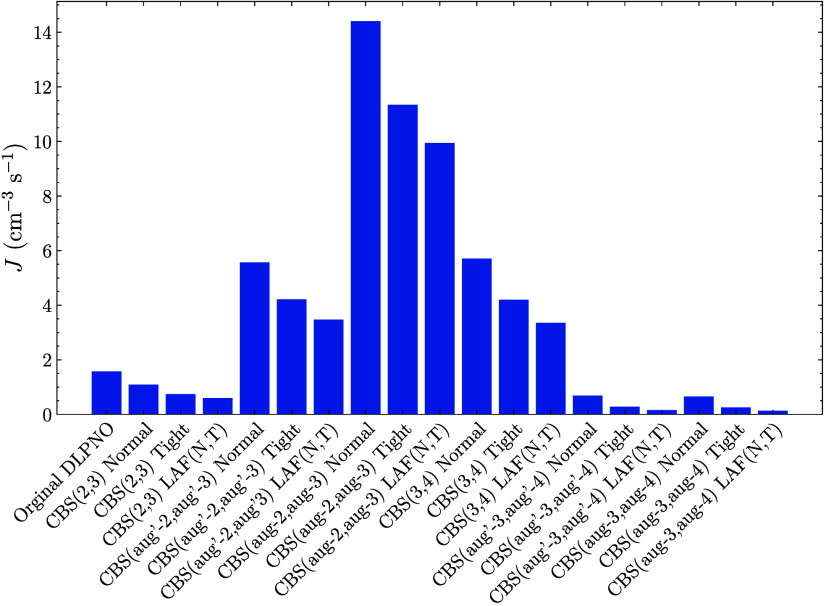
New particle formation rate as calculated by ACDC for the (SA)_1–4_(DMA)_1–4_ system with the single point at the given LNO–CCSD­(T) level given by the labels. The thermal contribution is calculated at the ωB97X-D/6-31++G­(d,p) level of theory. The temperature was set to 278.15 K, the SA concentration at 10^6^ molecules cm^–3^, and the DMA concentration at 10 ppt.

Lastly, to study the behavior of a loosely bound system, we simulated the NPF rates of the (SA)_1–4_(AM)_1–4_ system at 278.15 K, an SA concentration of 10^7^ molecules cm^–3^ and 10 ppb of AM (see Figure 8). For these conditions, the free energy is positive (at minimum 6 kcal/mol) for all methods, with the highest free energy cluster being the (SA)_3_(AM)_2_ cluster. Studying the possible growth paths, we find several barriers in the 5–7 kcal/mol range. Similarly to the lower concentration SA–DMA system, the rates drop with tighter locality settings and there is still a high variability for the rates. However, it should be noted that as the absolute NPF rates for the SA–AM system are relatively low, small differences between the methods are “insignificant” compared to other errors. Unlike the two previous examples, the CBS­(aug′-2,aug′-3) extrapolation and CBS­(3,4) no longer give the same rates, and the CBS­(3,4) instead follows the CBS­(aug-2,aug-3) rates. However, similar to before, the CBS­(aug′-3,aug′-4) and CBS­(aug-3,aug-4) extrapolations yield quite similar rates. This illustrates the robustness of these extrapolation procedures.

**Figure 8:**
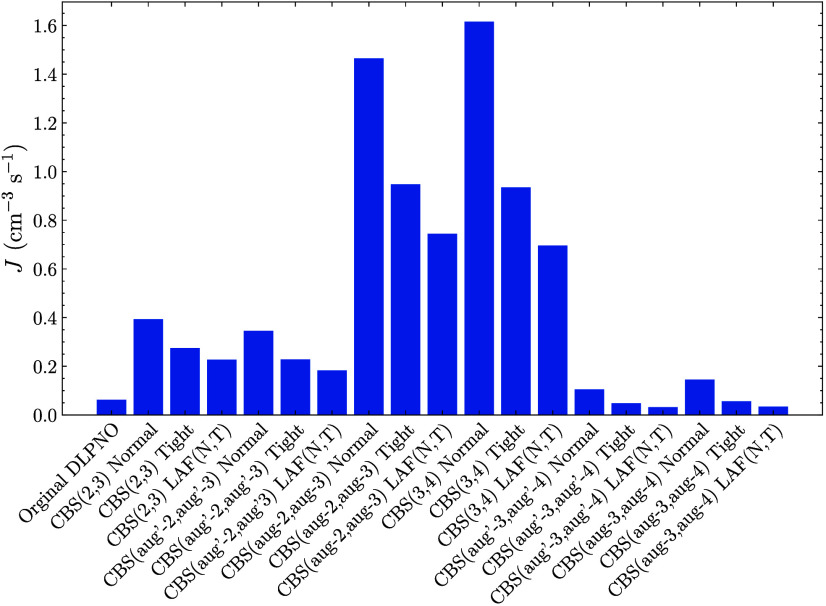
New particle formation rate as calculated by ACDC for the (SA)_1–4_(AM)_1–4_ system with the single point at the given LNO–CCSD­(T) level given by the labels. The thermal contribution is calculated at the ωB97X-D/6-31++G­(d,p) level of theory. The temperature was set to 278.15 K, the SA concentration at 10^7^ molecules cm^–3^, and the AM concentration at 10000 ppt.

Finally, to compare the rates of the wave function-based methods to commonly used DFT methods, we chose to test single-point energies for the SA–DMA system at the ωB97–3c, ωB97X-D3BJ/6-311++G­(3df,3pd), PW91/6-311++G­(3df,3pd), and M06-2X/6-311++G­(3df,3pd) levels of theory. For conditions matching the low SA concentration regime, the simulations yield rates of 0.13, 7.02, 5 × 10^–15^, and 0.02 cm^–3^ s^–1^, respectively. Except for the PW91 method, this is fairly decent accuracy compared to the DLPNO–CCSD­(T_0_)/aug-cc-pVTZ value of 1.57 molecules cm^3^ s^–1^. Hence, one would assume that DFT could potentially reveal the correct trends, but the absolute value could be slightly off.

From this section, we can conclude that for systems where the formation of the initial dimer cluster is rate-determining, the recommended methodology for calculating the binding energies follows the benchmark results in section 3.2. However, for systems where larger clusters are rate-determining, an increased sensitivity to the locality setting is observed, with decreasing NPF rates with a tighter locality setting. This indicates that the larger clusters’ local approximation error is more prominent. Hence, for studies on systems containing up to eight monomer clusters and extreme accuracy is warranted, we suggest the LAF­(N,T) LNO–CCSD­(T)/CBS­(aug-3,aug-4) level for calculating the binding energies, as it should, in principle, be closest to CCSD­(T)/CBS. However, the error in the thermal contribution is likely larger, and additional computational effort should perhaps be put toward reducing it instead.

### 3.5 Toward Accurate Binding Energies of Freshly Nucleated Particles

From the previous sections, it is clear that the LNO methods are a convenient choice for modeling large clusters. We are currently pushing the boundaries for the sizes of atmospheric molecular clusters we can accurately model. This has led to (SA)_*n*_(base)_*n*_ clusters with *n* up to 15 being sampled. However, for our previous studies of such large clusters, we have been limited to DFT using B97–3c for calculating the binding energies, which poses a potential large source of error. One of the largest clusters studied to date is the (SA)_15_(TMA)_15_ cluster (300 atoms) as depicted in Figure 9.^102,103^

**Figure 9:**
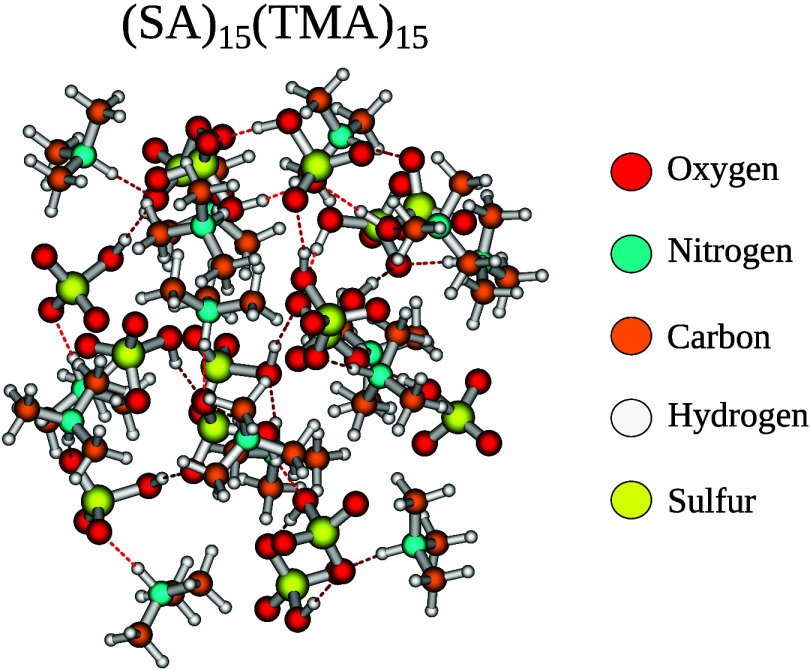
(SA)_15_(TMA)_15_ cluster at the B97–3c level of theory found by Wu et al.^102,103^

We find it possible to calculate the LAF­(N,T) LNO–CCSD­(T)/CBS­(aug′-3,aug′-4) level of theory for a cluster of this size. Comparing the electronic binding energy to the original B97–3c level of theory, we find that B97–3c yields a value of −748.2 kcal/mol compared to −778.0 kcal/mol at the LAF­(N,T) LNO–CCSD­(T)/CBS­(aug′-3,aug′-4) level. This massive 29.8 kcal/mol difference implies that the previously calculated binding energies are underbinding. This work illustrates that high-accuracy single points are now attainable even for very large clusters. If an even higher level of theory is needed, this should be attainable with machine learning methods. We suggest Δ-ML with kernel ridge regression (KRR) as we have previously shown that it can easily yield sub kcal/mol errors when trained on few conformers.^139–141^ However, if more data is available, the PaiNN architecture^142^ could also reach similar errors.^143^ In addition, KRR has been shown to be able to extrapolate well to larger cluster sizes.^100,141^ Hence, we can train Δ-ML KRR model on smaller (acid)_1–15_(base)_1–15_ clusters and accurately predict the binding energies of even larger clusters than previously studied. This will enable hitherto unseen accuracy of the binding energies of large atmospheric molecular clusters. Another possibility could be to employ cluster-in-molecule (CIM) or similar fragmentation approaches.^144^

## 4 Conclusions

We have constructed an extended test set of RI-MP2/aug-cc-pVTZ equilibrium structures of all possible monomers and dimers (except double base structures) of sulfuric acid (SA), formic acid (FA), nitric acid (NA), methanesulfonic acid (MSA), water (W), ammonia (AM), methylamine (MA), dimethylamine (DMA), trimethylamine (TMA), and ethylenediamine (EDA). This leads to a versatile testset of 218 cluster structures.

We benchmark the local coupled cluster methods, DLPNO–CCSD­(T_0_) and LNO–CCSD­(T), on the test set using different basis sets and extrapolation procedures to the complete basis set (CBS), local approximation free (LAF), and complete PNO space (CPS) limits. The extrapolations are tested against the binding energies of high-level CCSD­(F12*)­(T+)/cc-pVTZ-F12 reference calculations.

Five settings were probed for the DLPNO methods: NormalPNO with the PNO threshold set from 2 orders of magnitude lower to 2 orders of magnitude higher. Six settings for the LNO methods: vvLoose to vvTight. The basis sets tested were cc-pV*X*Z, aug′-cc-pV*X*Z (no augmentation on hydrogen), and aug-cc-pV*X*Z (*X* = D,T,Q).

Using the augmented basis sets with the DLPNO method, we find that the commonly applied DLPNO–CCSD­(T_0_)/aug-cc-pVTZ results rely on the cancellation of errors between the basis set error and the locality error to give an error distribution centered around zero. This ideal cancellation only happens for the LNO method with the larger aug-cc-pVQZ basis set. However, the error distribution is much narrower for the LNO methods at the larger basis set sizes compared to the DLPNO method, and we find the cost-to-accuracy ratio to be better as well. We therefore chose to focus on the LNO methods. In general, we also find the error to be fairly insensitive to the locality settings and extrapolations thereof for the LNO methods on the dimer test set.

Testing the reduced augmented basis set (everything but H is augmented), we find the error measures to be higher than the equivalent locality setting for the augmented basis set, However, the error distribution is still narrow, and the slight increase in error might be worth the gain in computational efficiency.

For the nonaugmented basis sets, we find that the CBS­(3,4) extrapolation performs excellently and is nearly identical with the fully augmented basis set results. This indicates that the Normal LNO–CCSD­(T)/CBS­(3,4) level of theory might be a sweet spot between accuracy and computational cost for calculating the binding energies of (smaller) atmospheric molecular clusters. However, as this might not hold for larger clusters, we suggest doing the LNO–CCSD­(T)/CBS­(aug-3,aug-4) calculation at at least the Normal setting for the usually studied cluster sizes of 2–8 monomers.

Using the Atmospheric Molecular Dynamics Code, we simulated the new particle formation rate of the (SA)_1–4_(AM)_1–4_ and (SA)_1–4_(DMA)_1–4_ systems with different basis set extrapolation, the Normal and Tight locality settings and extrapolation of them. We find an increased sensitivity to the locality settings for larger clusters compared to the dimers, but the basis set error is still the most dominant; i.e., the rates would benefit from doing an LAF extrapolation, but the thermal contribution is likely a much larger source of error at this point.

Finally, we show for the large (SA)_15_(TMA)_15_ cluster (300 atoms), that it is possible to perform the CBS extrapolation using aug′-cc-pVTZ and aug′-cc-pVQZ together with the LAF extrapolation using the Normal and Tight settings for the LNO–CCSD­(T) method. This illustrates that employing the LNO methods, high-accuracy single points are now attainable even for very large clusters.

## Supplementary Material





## Notes

Structures and energies (ZIP)
Table with the number of structures; The DLPNO cc-pV*X*Z and aug′-cc-pV*X*Z figures; Link to the Atmospheric Molecular Cluster Database with structures, log files, and spreadsheets of the properties (PDF)
