# Supplementary material for: Extrapolating Local Coupled Cluster Calculations toward CCSD(T)/CBS Binding Energies of Atmospheric Molecular Clusters
Source: ACS Omega. 2025 Sep 29;10(40):46794–808. doi: 10.1021/acsomega.5c04476 (PMC12529178; doi:10.1021/acsomega.5c04476)
Supplement: Supplementary file 2 [file ao5c04476_si_002.pdf]

Supporting Information: Extrapolating Local Coupled Cluster Calculations Toward CCSD(T)/CBS Binding Energies of Atmospheric Molecular Clusters

Yosef Knattrup and Jonas Elm*

Department of Chemistry, Aarhus University, Langelandsgade 140, 8000 Aarhus C, Denmark

E-mail: jelm@chem.au.dk

Phone: +45 28938085

S1 Number of Conformers

Table S1: Number of conformers for each cluster in the test set.

Cluster	Number of conformers
$(FA)_1(EDA)_1$,	32
$(SA)_1(EDA)_1$	30
$(MSA)_1(EDA)_1$	17
$(NA)_1(EDA)_1$	17
$(SA)_1(FA)_1$	13
$(SA)_1(NA)_1$	13
$(SA)_1(MSA)_1$	12
$(SA)_2$	11
$(FA)_1(W)_1$	7
$NA)_1(FA)_1$	7
$(SA)_1(DMA)_1$	5
$(FA)_2$	5
$(FA)_1(DMA)_1$	4
$(MSA)_1(FA)_1$	4
$(MSA)_1(W)_1$	4
$(SA)_1(W)_1$	4
$(FA)_1(TMA)_1$	3
$(MSA)_1(DMA)_1$	3
$(MSA)_1(NA)_1$	3
$(SA)_1(TMA)_1$	3
$(FA)_1(MA)_1$	2
$(MSA)_1(MA)_1$	2
$(MSA)_1(TMA)_1$	2
$(NA)_1(DMA)_1$	2
$(NA)_1(W)_1$	2
$(SA)_1(AM)_1$	2
$(MSA)_2$	2
$(NA)_2$	2
$(FA)_1(AM)_1$	1
$(MSA)_1(AM)_1$	1
$(NA)_1(AM)_1$	1
$(NA)_1(MA)_1$	1
$(NA)_1(TMA)_1$	1

S2 DLPNO Results

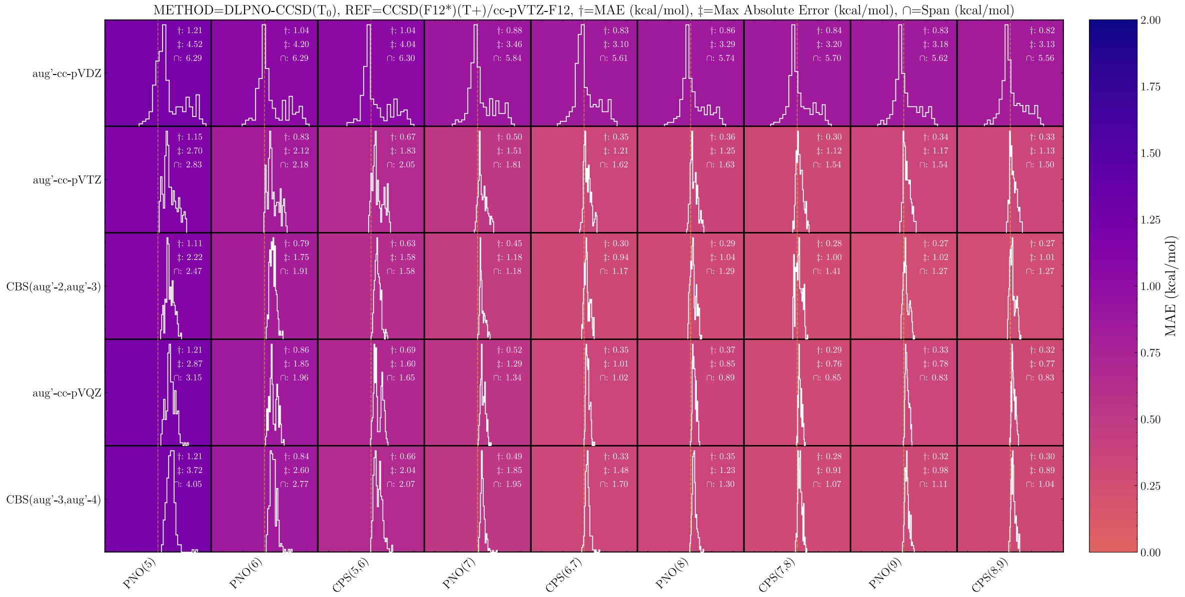

Figure S1: The binding energy errors of the DLPNO method using the reduced augmented basis sets compared to CCSD(F12*)(T+)/cc-pVTZ-F12. The PNO() labels indicate calculations performed at NormalPNO settings with the PNO thresholds set using the number as the exponent. CPS(,) and CBS(,) denote complete PNO space and complete basis set extrapolations, respectively. Each box spans 10 kcal/mol. Negative values (left) indicate overbinding relative to the reference method.

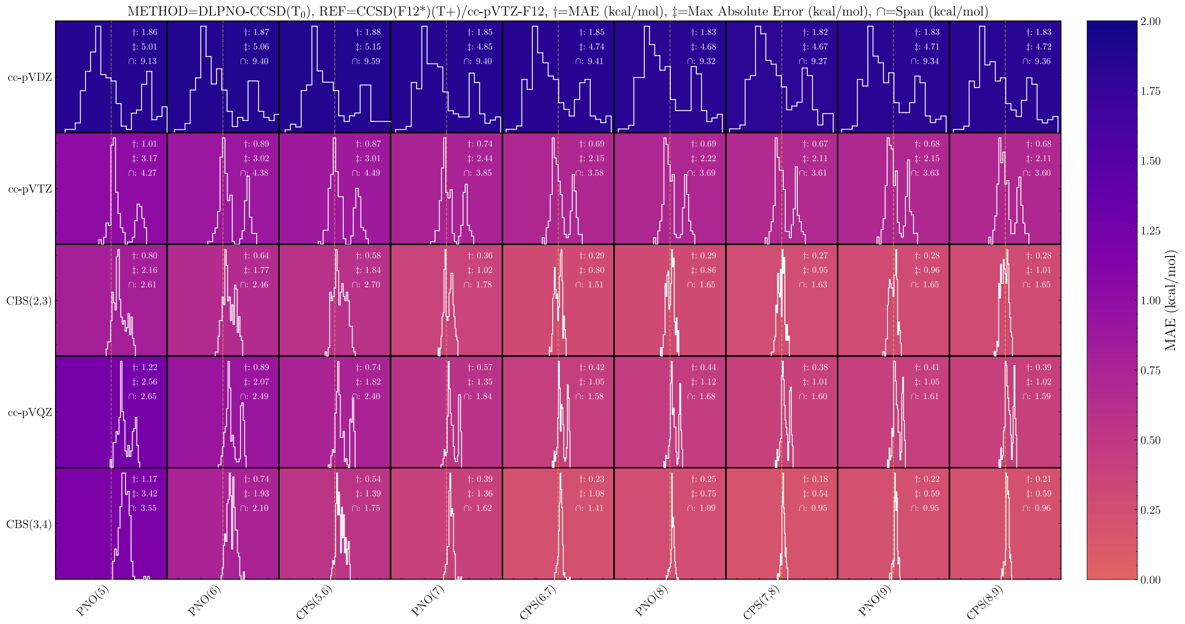

Figure S2: The binding energy errors of the DLPNO method using the non-augmented basis sets compared to CCSD(F12*)(T+)/cc-pVTZ-F12. The PNO() labels indicate calculations performed at NormalPNO settings with the PNO thresholds set using the number as the exponent. CPS(,) and CBS(,) denote complete PNO space and complete basis set extrapolations, respectively. Each box spans 10 kcal/mol. Negative values (left) indicate overbinding relative to the reference method.

S3 Database

All structures, log files, csv files with energies, and the csv file with free energies under given conditions are freely available in the Atmospheric Molecular Cluster Database:

`https://github.com/elmjonas/ACDB/tree/master/Articles/knattrup25_local_benchmark`
